# Loss to follow‐up in the hepatitis C care cascade: A substantial problem but opportunity for micro‐elimination

**DOI:** 10.1111/jvh.13399

**Published:** 2020-09-22

**Authors:** Marleen van Dijk, Joost P.H. Drenth, the HepNed study group, Joop E Arends, Sylvia M Brakenhoff, Cas J Isfordink, Rob de Knegt, Marc van der Valk

**Affiliations:** ^1^ Department of Gastroenterology and Hepatology Radboud University Medical Centre Nijmegen the Netherlands; ^2^ Department of Infectious Diseases University Medical Centre Utrecht Utrecht the Netherlands; ^3^ Department of Gastroenterology and Hepatology Erasmus Medical Centre Rottedam the Netherlands; ^4^ Division of Infectious Diseases Amsterdam Infection & Immunity Institute Amsterdam University Medical Centre Amsterdam the Netherlands; ^5^ Department of Gastroenterology and Hepatology University Medical Centre Utrecht Utrecht the Netherlands

**Keywords:** cascade of care, hepatitis C, lost to follow‐up, micro‐elimination

## Abstract

Since the advent of direct‐acting antivirals, elimination of hepatitis C viral (HCV) infections seems within reach. However, studies on the HCV cascade of care show suboptimal progression through each step for all patient groups. Loss to follow‐up (LTFU) is a major issue and is a barrier to HCV elimination. This review summarizes the scale of the LTFU problem and proposes a micro‐elimination approach. Retrieving LTFU patients and re‐engaging them with care again has shown to be feasible in the Netherlands. Micro‐elimination through retrieval can contribute to reaching the World Health Organization's viral hepatitis elimination targets by 2030.

## 1 BACKGROUND

The global hepatitis C virus (HCV) epidemic stimulated the World Health Organization (WHO) to develop viral hepatitis elimination targets in 2016.^1^ An estimated 71 million people worldwide were infected by HCV in 2015.^2^ Thus, the WHO set the target of a 90% reduction in new infections and a 65% reduction in viral hepatitis‐related mortality by 2030 as compared to 2015. These are ambitious but feasible goals, since we have ample tools at hand to curtail the current HCV epidemic. The diagnosis of active HCV can be readily made, by means of sample analysis in a central facility or through point‐of‐care testing. Direct‐acting antivirals (DAAs) cure the infection in ≥95% of cases.^3^ Pangenotypic DAAs can be used in all patients with only a few barriers such as potential drug‐drug interactions or presence of (decompensated) cirrhosis.^4^, ^5^ Most countries have assessed their specific HCV population and the availability of tools in their countries and subsequently developed national hepatitis plans in line with the WHO elimination targets.^6^

HCV elimination according to the WHO goals can be achieved in various ways, which ideally should be incorporated in a multifaceted approach. We can focus on prevention, by developing a vaccine or by increasing awareness and educating groups at risk of transmission of the virus. Secondly, we can develop or augment existing screening strategies, in order to diagnose more patients. Lastly, we can treat as many infected patients as possible. Since the development of highly effective and tolerable DAAs, HCV elimination projects have primarily focused on prevention and screening, since treatment was not seen as a problem anymore. However, ensuring treatment for all diagnosed patients remains a problem to this day.

Loss to follow‐up (LTFU) prevents patients from receiving the care they need to be cured of their infection. The extent of this problem remains unclear, especially in the DAA era. In order to grasp the scope of the LTFU problem, one needs to understand the HCV care cascade and how patients move through its phases. This review aims to assess published literature on LTFU in the HCV cascade of care during the DAA era and will provide an overview of issues and possible solutions.

## 2 CONCEPT OF LOSS TO FOLLOW‐UP

Different definitions for LTFU are used in the literature, since patients become lost to follow‐up for various reasons. For example, they may have moved house, emigrated, died or been imprisoned. Many times, the reason for LTFU cannot be ascertained as contact with the patient cannot be established. Retrospective observational studies often do not provide a specific definition or use nonattendance to any appointment as a definition.^Suppl file 1‐11^ Some of these studies mention death separately and do not include this as a reason for LTFU.^Suppl file 12‐17^ Other studies that reviewed ever‐diagnosed patients defined LTFU as patients who never or not recently had an appointment with an HCV specialist.^Suppl file 18,19^ Interventional studies aiming to improve the cascade of care also do not give a definition or define LTFU as nonattendance anywhere in the care cascade,^Suppl file 20‐49^ often separating death from the LTFU group.^Suppl file 50‐58^ There are some studies that include multiple LTFU definitions and report data on all of them, such as nonattendance, nonresponse to invitation, moved, incarcerated, no insurance and comorbidities.^Suppl file 59‐62^

There may be a lesson to be learned on defining LTFU from studies in other fields of medicine. Previously mentioned HCV studies did not take time into account when defining LTFU. Prospective studies defined LTFU as nonattendance at the end of their study period, which varied greatly among studies. Retrospective studies defined LTFU as nonattendance since their last visit up to study initiation. HIV studies have investigated LTFU extensively and showed that the way you define LTFU greatly influences your LTFU outcomes.^7^ In addition, these studies have demonstrated different ways to determine the ideal timeframe to classify someone as LTFU, that is x days after last clinic visit.^8^, ^9^ When different studies use different definitions, it is virtually impossible to compare care cascades and combine results. However, since this is the case for the HCV studies assessed in this review, we chose a pragmatic approach that suits the illustrative purpose of this review. We define LTFU as nonattendance to any appointment in the care cascade at any time since their last visit. Patients who had died were not included in the definition of LTFU.

## 3 THE HCV CARE CASCADE

**FIGURE 1:**
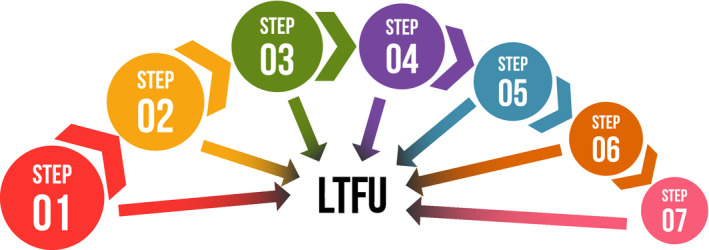
Hepatitis C care cascade. Step 1: HCV prevalence; step 2: diagnosed with chronic HCV; step 3: linked to care; step 4: liver disease assessed; step 5: started on treatment; step 6: achieved SVR; step 7: accessed chronic post‐SVR care. Figure freely adapted with permission from Safreed‐Harmon et al.^12^ HCV, hepatitis C virus; LTFU, lost to follow‐up; SVR, sustained virological response

In order to grasp the magnitude of the LTFU problem in chronic HCV patients, we must first understand the HCV care cascade. Reviewing published literature on this subject shows that definitions of the care cascade vary with each paper. However, efforts to come up with an unambiguous description of the HCV care cascade have been made. In 2018, the WHO established a monitoring framework that includes 10 core indicators addressing prevention, diagnosis, treatment and mortality.^10^ The WHO states that four of the 10 core indicators should be used for cascade of care reporting: the number of patients infected, diagnosed, treated and cured.^11^ Recently, a study group comprised of clinical, epidemiological and public health experts from Australia, Europe and North America have proposed a clarified and slightly extended care continuum.^12^ Their Consensus HCV Cascade of Care (CHCoC) is based on the WHO indicators, a review of published literature on HCV care continuums and on methodological issues in HIV cascade of care monitoring. It can be divided into four key steps (the four WHO indicators) and three supplementary steps: (a) estimated HCV prevalence; (b) diagnosed with chronic HCV; (c) linked to HCV care; (d) liver disease assessed; (e) started on treatment in (year); (f) achieved sustained virological response (SVR) in (year); and (g) accessed chronic post‐SVR care. The authors provided pragmatic definitions for the four key steps, which stakeholders can use to report on elimination progress. Understandably, by increasing the number of steps in the care cascade, the chances of being lost from care also increase. LTFU is seen as a major problem, because it remains unsure whether the patient is cured or not. Liver disease in these patients may progress, and they may even contribute to HCV transmission if they still exhibit certain risk behaviour. When reviewing the literature published on HCV care cascades in the DAA era and their LTFU rates, we used the CHCoC to report our findings (see Figure 1). And overview and characteristics of the included studies in this review can be found in Table 1.

**TABLE 1:** Characteristics of studies included in review on the HCV care cascade in mixed populations, people who inject(ed) drugs and HIV/HCV‐coinfected patients.

References^a^	Country	Summary	Percentage of loss to follow‐up in each CHCoC step, including our definition					Intention to treat sustained virological response^b^
			CHCoC step 2: HCV RNA not assessed in anti‐HCV positive patients	CHCoC step 3: absence at follow‐up appointment after diagnosis/ referral	CHCoC step 4: liver disease not assessed in diagnosed/ attendees	CHCoC step 5: treatment not initiated in attendees	CHCoC step 6: LTFU during or after treatment	
*Mixed population*								
Zucker, 2018^1^	USA	Retrospective analysis of anti‐HCV positive patients in academic hospital diagnosed in DAA era, using an electronic medical record algorithm	28%	73%^c^		70%		39%
Assoumou, 2020^2^	USA	Retrospective analysis of multicentre FQHC cohort	27%		48% (APRI)	88%		31%
Al‐Khazraji, 2020^3^	USA	Retrospective analysis of HCV positive patients in academic hospital diagnosed in DAA era who are eligible for treatment (RNA‐positive and no comorbidities with short life expectancy)				80% (67% of these were confirmed LTFU)	5.3%	
Moore, 2018^4^	USA	Retrospective analysis of microbiology database (mandated reporting of positive HCV tests)				48%		65%
Nguyen, 2017^5^	USA	Retrospective analysis of RNA‐positive patients seen in academic clinic in the DAA era				23% (24% of these were confirmed LTFU)		89%
Marshall, 2018^6^	USA	GT1 patients who initiated treatment at outpatient clinic care of an academic centre					7% (during) 15% (after)	74%
Haridy, 2018^12^	Australia	Observational, prospective study of all treated patients in (association with) tertiary centres (including prisons and community health centres via remote consultation)			32% (Fibroscan) (community‐based vs hospital 43% vs 30%)		14.7%	80%
Sølund, 2018^13^	Denmark	Analysis of the Danish Database for Hepatitis B and C, including HCV patients eligible for treatment				51% (of these, 30% were LTFU)	1.5% (during) 3.2% (after)	88%
Darvishian, 2020^14^	Canada	Multicentre cohort study of GT1 and 3 patients, treated by specialists or GPs					8%	
Scaglione, 2020^16^	Italy	Retrospective analysis of all treated HCV patients in a teaching hospital					10%	87%
Adamson, 2020^18^	USA	Retrospective cohort study comparing treatment by HCV specialists in a primary care practice to treatment by HCV specialists in hospitals	93% (of combined cohort)	25% vs 52%		58% vs 53%		25% vs 22%
Dever, 2017^19^	USA	HCV patients from the Veteran Affairs HCV registry with increased risk of advanced fibrosis that never attended an appointment or were LTFU were retrieved		45%		26% (54% of these were confirmed LTFU)		
Trooskin, 2015^20^	USA	POC testing (anti‐HCV and RNA) in community‐based settings, positive patients counselled and referred by patient navigator	13%	9%	5% (liver ultrasound, HepaScore^d^ or FibroSure^e^	43%		
Coyle, 2019^21^	USA	Implementation of routine HCV testing and linkage to care in five FQHCs, including medical assistant‐initiated testing, automated health record prompts, reflex testing and care coordinators	4%	16%	20% (liver fibrosis panel, liver biopsy, liver ultrasound or Fibroscan)	78%		53%
Bajis, 2019^22^	Australia	Liver health promotion campaign and noninvasive fibrosis assessment followed by RNA screening and linkage to care among homeless in a community centre	1%	38% (100% of these were confirmed LTFU)	0% (Fibroscan)	21%		65%
Waked, 2020^23^	Egypt	Reported progress of the Egypt HCV elimination programme	33%			8%		82%
Khalid, 2020^24^	Pakistan	Decentralised screening and treatment including POC testing for people ≥1 risk factor in primary health clinic and treatment free of charge	82%		1% (APRI)	84%		
Hsieh, 2019^25^	USA	Known chronic HCV patients who visited the ED were offered linkage to care		66%	5% (FibroSure or Fibroscan)	58% (27% of these were confirmed LTFU)		94%
Zuckerman, 2018^26^	USA	Decentralised treatment by pharmacist‐led multidisciplinary team		27%	12% (ultrasound, liver biopsy, FIB‐4 or FibroSure)	17% (4% of these were confirmed LTFU)	5% (during) 4% (after)	88%
Evans, 2018^27^	United Kingdom	Opt‐out HBV and HCV screening and linkage to care for people ≥16 years at the ED, including reflex testing		22% (100% of these were confirmed LTFU)	40% (Fibroscan)	50% (44% of these were confirmed LTFU)		80%
Benitez, 2020^28^	USA	One‐time testing according to CDC guidelines and treatment in FQHCs and satellite centres serving a predominantly homeless population, including reflex testing		15%		84%	3.4% (during) 13% (after)	83%
Capileno, 2017^33^	Pakistan	Decentralised screening and treatment including POC testing for people ≥1 risk factor in primary health clinic and treatment free of charge			13% (APRI)	81%	4.7% (during) 3% (after)	83%
Cooper, 2017^34^	Canada	Retrospective analysis of patients treated at outpatient clinic compared to patients mainly treated through telemedicine			61% vs 84% (liver biopsy) 38% vs 41% (Fibroscan)	72% vs 83%		
Shiha, 2018^35^	Egypt	Free screening and treatment in rural village	0%		0% (Fibroscan)	4%		98%
Ford, 2017^36^	USA	Decentralised screening and linkage to care in FQHCs and addiction care services, treatment both on‐ and off‐site			52%	45%		91%
Bartholomew, 2019^37^	USA	Decentralised treatment in primary care by physician assistants and primary care physicians				30%	3.8% (during) 10% (after)	77%
Wade, 2018^38^	Australia	Remote consultation by specialists for GPs, treatment by GPs or after referral to specialist				29%		
Mendizabal, 2019^39^	Argentina	Tele‐mentoring of primary care physicians and specialists by a multidisciplinary team of specialists at an academic centre (ECHO), compared to treatment in tertiary centre				13% (in entire cohort)		68% vs 72%
Norton, 2017^42^	USA	Patients treated by specialist and HCV care coordinator in a FQHC					0% (during) 2.2% (after)	96%
Kattakuzhy, 2017^43^	USA	Nonrandomized trial comparing decentralised treatment by nurse practitioners, GPs or specialists in community health centres					2% vs 2.5% vs 3.5% (during) 4% vs 5% vs 4.8% (after)	89% vs 87% vs 84%
Carvalho‐Louro, 2020^50^	Brazil	Free POC testing for people >40 years old visiting laboratories	32% (100% of these were confirmed LTFU)		12% (Fibroscan)	29%	0% (during)	92%
Averhoff, 2020^51^	Georgia	Reported progress of the Georgia HCV elimination programme	20%			21%	0.4% (during) 25% (after)	66%
Hutton, 2019^52^	Australia	Screening and linkage to care of patients presented at ED with ≥1 risk factor, including POC testing	10%	67% (85% of these were confirmed LTFU)		0%	0% (during) 0% (after)	70%
Chiong, 2019^53^	Australia	Screening and linkage to care of inpatients in a tertiary hospital			23% (Fibroscan)	15% (50% of these were confirmed LTFU)	6.5% (during) 4.3% (after)	80%
Koren, 2019^55^	USA	Retrospective analysis of a pharmacist‐driven multidisciplinary treatment model					1.8% (during) 5.5% (after)	86%
Nouch, 2018^56^	Canada	Decentralised multidisciplinary care in community health centres					3.6% (during) 9% (after)	86%
White, 2019^57^	Australia	Decentralised treatment by primary care physicians or in secondary care					4.3% (during)	89%
McMahon, 2019^59^	USA	Analysis of the cascade of care for Alaskan Natives tested anti‐HCV positive via hepatitis programme	3%	37% (83% of these were confirmed LTFU)		23%		
Sherbuk, 2019^60^	USA	HCV patients treated in tertiary centre with dedicated nurse coordinator		24% (47% of these were confirmed LTFU)		20%	19% (after)	74%
Francheville, 2018^61^	Canada	Province‐wide model of care with centralized referral, triage and intake by a nurse coordinator, treatment by specialist				24% (0% of these were confirmed LTFU)		88%
Mohsen, 2019^62^	Australia	Tele‐mentoring of primary care physicians and specialists by a multidisciplinary team of specialists at an academic centre (ECHO), compared to treatment in tertiary centre				22% (36% of these were confirmed LTFU) vs 19%	0% vs 11% (during) 12% vs 3% (after)	77% vs 83%
*People who inject(ed) drugs*								
Christensen, 2018^7^	Germany	Analysis of the German hepatitis C registry, comparing former/current drug users on OST with former/current drug users not on OST and people with no documented drug use					2.7% vs 2.0% vs 0.7% (during) 7.6% vs 6.5% vs 2.5% (after)	85% vs 86% vs 92%
Falade‐Nwulia, 2020^29^	USA	Peer‐promoted screening and linkage to care in a PWID population, including incentives for testing	6%	64%		80% (66% including patients who were already linked to care)		
Bajis, 2020^30^	Australia	Liver health promotion campaign and non‐invasive fibrosis assessment followed by RNA screening and linkage to care in addiction care services		51%	0% (Fibroscan)	47% (23% including patients who were already linked to care)		
Alimohammadi, 2018^31^	Canada	Randomized controlled trial about decentralised treatment in community pop‐up clinics for PWID through directly observed treatment, compared to group therapy and standard care, including POC testing and incentives		50%		39%	6% (after)	85%
Harrison, 2019^32^	United Kingdom	Multicentre study on increasing screening and linkage to care in addiction care services		35% (26% of these were confirmed LTFU)		66% (6% of these were confirmed LTFU)		82%
Wade, 2020^40^	Australia, New Zealand	Randomized controlled trial on treatment of PWID in primary care facilities that provide OST, compared to treatment in hospital			21% vs 38% (Fibroscan)	10% (40% of these were confirmed LTFU) vs 38% (45% of these were confirmed LTFU)	4.7% vs 5.6% (during) 28% vs 6% (after)	
O’Sullivan, 2020^41^	United Kingdom	Decentralised screening and treatment in a nurse‐led programme in addiction care services, including reflex testing	0%		13% (Fibroscan)	52% (0% of these were confirmed LTFU)	4.3% (after)	90%
Morris, 2017^44^	Australia	Decentralised care including care coordinators for patients in addiction medicine					2% (during) 20% (after)	80%
Read, 2017^54^	Australia	Decentralised treatment in primary care facility targeted to PWID					2.8% (during) 12.5% (after)	82%
Koustenis, 2020^58^	Greece	Retrospective analysis of single centre PWID cohort treated in tertiary centre					2.3% (during) 10.3% (after)	80%
*HIV/HCV‐coinfected population*								
Falade‐Nwulia, 2019^8^	USA	Retrospective analysis of all HIV/HCV‐coinfected patients in an academic hospital		5%		18%	8.8% (after)	87%
Saris, 2017^9^	Netherlands	Retrospective analysis of all HIV/HCV‐coinfected patients from two hospitals (one academic), planned for DAA treatment			19% (Fibroscan)	9% (none LTFU)		80%
Kronfli, 2018^10^	Canada	Retrospective analysis of multicentre HIV/HCV‐coinfected cohort (hospitals, community‐based clinics and street outreach programmes)				64% (28% of these were confirmed LTFU)		
Adekunle, 2020^11^	USA	Retrospective analysis of HIV/HCV‐coinfected hospital cohort		10%		10% (8% of these were confirmed LTFU)	0%	96%
Cachay, 2019^15^	USA, Spain, Italy	Retrospective analysis of all HIV/HCV‐coinfected patients treated in five hospitals in three countries					1%	93%
Starbird, 2020^47^	USA	Randomized controlled trial where HIV/HCV‐coinfected patients who were not in HCV care would receive usual care or nurse case management focusing on linkage to care		75% vs 53%		0% vs 75% (11% of these were confirmed LTFU)		
Ward, 2019^48^	USA	Randomized controlled trial of screening and linkage to care of HIV/HCV‐coinfected patients, comparing usual care (nurse‐led multidisciplinary team including incentives for study‐specific visits) with usual care including peer support and usual care including incentives. Treatment was free of charge				24% (33% vs 17% vs 24%)	2.7% (during; 4.2% vs 2.2% vs 2.4%) 0% (after)	91% (92% vs 91% vs 90%)
Rizk, 2019^49^	USA	Retrospective analysis of a single centre HIV/HCV‐coinfected cohort treated by a dedicated multidisciplinary team		9%		17%		

CDC, Centers for Disease Control and Prevention; CHCoc, Consensus HCV Cascade of Care; DAA, direct acting antiviral; ED, emergency department; FQHC, federally qualified health centre; GP, general practitioner; GT, genotype; HCV, hepatitis C virus; LTFU, loss to follow‐up; OST, opioid substitution therapy; POC, point of care; PWID, people who inject drugs; USA, United States of America.

^a^ In Supplementary file 1.

^b^ Defined as SVR among those who initiated therapy.

^c^ From this step on, patients without a viral load assessment were included.

^d^ HepaScore uses the patient’s age, sex and bilirubin, γ‐glutamyl transferase, α2‐macroglobulin and hyaluronic acid levels to determine fibrosis stage.

^e^ FibroSure uses the patient’s age, sex and ALT, bilirubin, γ‐glutamyl transferase, α‐2 macroglobulin, haptoglobin and apolipoprotein A1 levels to determine fibrosis and necroinflammatory stage.

## 4 LTFU DURING DIAGNOSTIC ASSESSMENT (CHCOC STEP 2)

The first step in diagnosing chronic HCV is the determination of presence of HCV antibodies. However, the key step in confirming the diagnosis of chronic HCV is determining HCV RNA (or HCV core antigen when RNA assays are not available or not affordable).^13^ In many countries, HCV RNA is not tested automatically after receiving a positive antibody test result. This two‐step diagnosis provides the first opportunity for patients to become LTFU. Two retrospective, observational studies have shown that approximately 72% of their anti‐HCV‐positive populations were tested for HCV RNA.^Suppl file 1,2^ This percentage is generally higher in interventional studies aiming to improve the cascade of care, often done in community‐based settings: 67%‐100% (median 90%).^Suppl file 20‐23,50‐52,59^ However, some studies have shown that only 7%^Suppl file 18^ or 18%^Suppl file 24^ of anti‐HCV‐positive people receive confirmatory testing. Reasons for this vary and are often unreported, but might be due to LTFU. One study confirmed that 32% of anti‐HCV‐positive people were LTFU before receiving an RNA test.^Suppl file 50^ Reflex testing, where the laboratory automatically tests for HCV RNA or HCV core antigen when the antibody test proves to be positive, improves this step in the cascade of care.^Suppl file 27^

## 5 LTFU BEFORE LINKAGE TO HCV CARE (CHCOC STEP 3)

When someone has tested positive for HCV RNA, referral to an HCV specialist for further evaluation should follow. However, attendance to this follow‐up visit is only reported in 27%‐91% (median 68%) of cases.^Suppl file 1,18‐22,25‐28,52,59,60^ People who inject(ed) drugs (PWID), a well‐known hard to reach population, attend in 36%‐65% of cases (median 50%).^Suppl file 29‐32^ In HIV/HCV‐coinfected patients, attendance seems to be higher with 25%‐95% (median 90%).^Suppl file 8,11,47,49^ Generally speaking, attendance is higher for those under decentralized care. Reasons for absence are difficult to assess; however, some studies confirm LTFU in 26%‐100% (median 84%) of absentees.^Suppl file 22,27,32,52,59,60^

## 6 LTFU DURING LIVER DISEASE ASSESSMENT (CHCOC STEP 4)

Several diagnostic procedures are available to grade and stage liver disease. Where liver biopsy was standard of care in the past, nowadays noninvasive methods are largely preferred. Liver fibrosis may be quantified by using serological panels, such as the widely used FIB‐4 (using the patient's age, platelet count, AST and ALT levels) or APRI score (using AST levels, the AST upper limit of normal and platelet count), or by using transient elastography. Almost all studies in the DAA era employ noninvasive ways to assess liver disease severity. When looking at people who have attended their first visit after being diagnosed or referred, fibrosis was assessed with the APRI score^Suppl file 2,24,33^ in 52%‐99% (median 87%) and with FibroScan^Suppl file 9,12,22,25,27,30,34,35,40,41,50,53^ in 59%‐100% (median 79%). Studies which used other noninvasive measures or did not report which measures were used, reported assessment in 48%‐95% (median 88%) of attendees.^Suppl file 20,21,25,26,36^ LTFU may contribute to this suboptimal assessment rate and should be addressed.

## 7 LTFU BEFORE INITIATING TREATMENT (CHCOC STEP 5)

Even in the era of highly effective DAAs, treatment initiation rates are low. LTFU proves to be a large contributor to this problem. Retrospective studies have shown that only 12%‐77% (median 29%) of patients diagnosed or engaged in care during the DAA era initiated treatment after being diagnosed with chronic HCV.^Suppl file 1‐5,13,34^ Interventional studies aimed to improve the care cascade show that this rate can increase to 16%‐100% (median 73%).^Suppl file 18‐28,33,35‐39,50‐53,59‐62^ Studies in the HIV field show similar results, with 36%‐91% (median 90%) initiating treatment in retrospective studies^Suppl file 9‐11^ and 25%‐100% (median 80%) in interventional studies.^Suppl file 47‐49^ However, the treatment rate remains suboptimal in PWID with only 20%‐90% (median 53%) initiating treatment.^Suppl file 29‐32,40,41^ Generally, treatment initiation rates are higher in decentralized settings, both in PWID and non‐PWID populations. Reasons for poor treatment initiation rates vary. Unfortunately, many countries still experience restrictions in who can and cannot be treated with DAAs.^14^, ^15^ This problem may especially apply to studies from the first stages of the DAA era.^16^ Other reasons for poor treatment initiation rates may be comorbidities or perceived lack of compliance. However, LTFU contributes to a large extent to these poor rates. Studies showed that LTFU is the reason for nontreatment in 0%‐67% (median 33%) of cases.^Suppl file 3,5,13,19,25‐27,32,40,41,53,61,62^

## 8 LTFU DURING OR AFTER TREATMENT (CHCOC STEP 6)

As we know from registration trials, DAAs are highly effective. Real‐world studies yield similar results. However, LTFU influences result in real‐world studies significantly more. Recently, Darvishian et al showed that LTFU exceeded viral failure in their real‐world study, impeding the cascade of care. Studies show that 0%‐11% (median 3.4%) of patients become LTFU during therapy^Suppl file 6,13,26,28,33,37,42,43,50‐53,55‐57,62^ and that 0%‐25% (median 4.9%) become LTFU after therapy completion, with missing SVR values.^Suppl file 6,13,26,28,33,37,42,43,51‐53,55,56,60,62^ PWID show similar results with 0.7%‐5.6% (median 2.5%) becoming LTFU during treatment^Suppl file 7,40,44,54,58^ and 2.5%‐28% (median 7.1%) after.^Suppl file 7,31,40,41,44,54,58^

**FIGURE 2:**
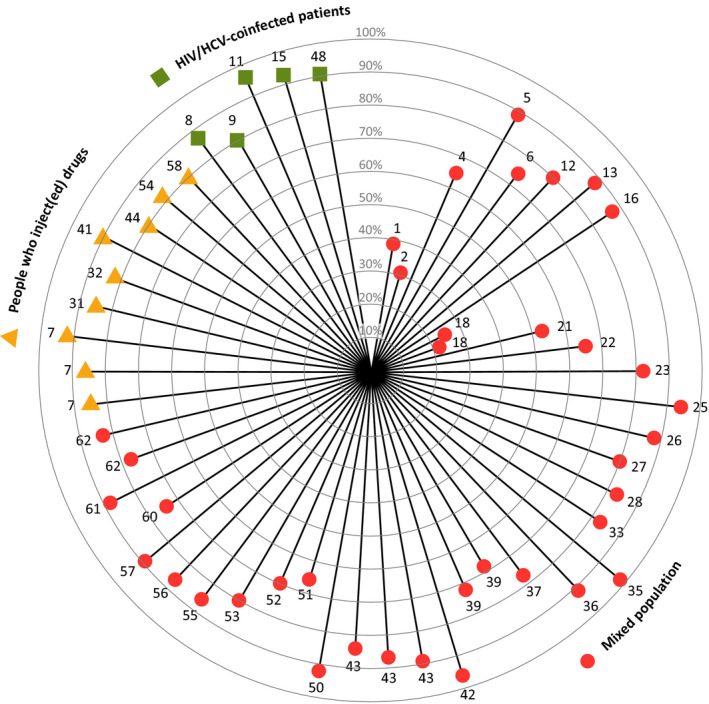
Intention‐to‐treat sustained virological response percentages in studies included in this review in mixed populations, people who inject(ed) drugs and HIV/HCV‐coinfected patients. Each line represents one study or one study group. The corresponding number refers to the reference in Supporting File 1

Many studies report intention‐to‐treat SVR percentages, defined as the proportion of patients who reached SVR out of the number of patients that initiated DAA therapy (see Figure 2). In studies including mixed populations, ITT SVR varies from 22% to 98% (median 83%).^Suppl file 1,2,4‐6,12,13,16,18,21‐23,25‐28,33,35‐37,39, 42,43,50‐53,55‐57,60‐62^ In studies focusing on PWID populations, ITT SVR ranges from 80% to 92% (median 85%).^Suppl file 7,29‐32,40,41,44,54,58^ Lastly, in studies focusing on HIV/HCV‐coinfected populations, ITT SVR ranges from 80% to 96% (median 91%).^Suppl file 8‐11,15,47‐49^

## 9 LTFU AFTER SVR (CHCOC STEP 7)

Guidelines suggest that people with advanced fibrosis (METAVIR score F3) or cirrhosis (F4) who reached SVR should be subjected to surveillance for hepatocellular carcinoma (HCC) every six months by means of ultrasound.^13^, ^17^ Furthermore, cirrhotic patients with varices present at pre‐treatment endoscopy should be surveyed for oesophageal varices.^13^, ^17^ Unfortunately, data on how many cured patients actually receive such surveillance are lacking. Most studies stop reporting on the care cascade at the moment SVR is reached. A recent review showed that less than 30% of cirrhotic patients are included in surveillance programmes, independent from aetiology.^18^ More studies on the surveillance adherence among cured HCV patients with an indication for surveillance are needed.

## 10 FACTORS ASSOCIATED WITH LTFU

Younger age (~45 and younger),^Suppl file 12,46,58,60^ treatment in hospital,^Suppl file 18^ a history of homelessness,^Suppl file 19,54^ mental illness^Suppl file 15,45^ and insurance type^Suppl file 26,60^ were some of the most common factors associated with LTFU. Factors associated with retention in care were older age (~60 and older)^Suppl file 14,16^ and HIV coinfection.^Suppl file 14,46^ However, one study with HIV coinfected patients showed that detectable HIV viral load was actually associated with LTFU,^Suppl file 45^ possibly reflecting suboptimal retention in HIV care. Studies on the HCV care cascade in people living with HIV confirm relatively good retention in care, especially after starting treatment.^Suppl file 8‐11,15,17,45,47‐49^ The last factors that were often associated with LTFU are linked to injecting drug use. Past,^Suppl file 14^ recent^Suppl file 7,14^ or ongoing drug use^Suppl file 15,19,45^ was mentioned in several studies as being associated with LTFU. Receiving opioid substitution therapy in one centre and DAA treatment in another was also associated with LTFU.^Suppl file 7,56^

## 11 MICRO‐ELIMINATION OF LTFU PATIENTS THROUGH RETRIEVAL

LTFU occurs in all steps of the care cascade and may severely impact HCV care and opportunities for cure. It is reasonable to assume that data from the interferon era on LTFU are worse, due to the fact that fewer patients had an indication for treatment, more patients refused treatment, fewer patients finished the ill‐tolerated treatment and only a limited number of patients achieved cure, compared to the DAA era. This hypothesis was confirmed in a recent study by Aleman et al^19^ The authors included HCV patients from their national register diagnosed between 2001 and 2011 and alive in 2013, and found that an impressive 61% was LTFU. A study from Belgium using a similar approach showed that PWID and patients who never received HCV treatment had a higher risk of becoming LTFU (OR 2.2 for both).^20^ This provides us with an opportunity as the LTFU HCV population may be an excellent candidate for micro‐elimination, the process of eliminating HCV in subpopulations.^21^ Micro‐elimination is the favoured approach in many countries, especially in those with a relatively low national prevalence, but higher prevalence in specific subpopulations. Lazarus et al have recently described which subpopulations should be considered for micro‐elimination, such as aboriginal and indigenous communities, HIV/HCV‐coinfected people, migrants from high‐prevalence countries, people who inject drugs, people with inherited blood disorders and prisoners.^22^ We propose that LTFU patients should be added to this list. As indicated, LTFU is a substantial problem across the entire care cascade. Because this HCV population has already been identified, it is obvious that retrieval of these patients can be considered ‘low‐hanging fruit’.

## 12 RETRIEVAL IN THE NETHERLANDS: THE CELINE PROJECT

Several regional projects have been executed in the Netherlands focused on the LTFU population. We found that up to 14% of our HCV population diagnosed in the previous 15 years was LTFU before being cured and eligible for retrieval.^23^, ^24^, ^25^ Based on best practices from these projects, a nationwide approach was developed.^26^, ^27^ The hepatitis C Elimination in the Netherlands (CELINE) project aims to retrieve LTFU chronic HCV patients and re‐engage them with care. The protocol is described in detail elsewhere.^27^ In short, we identify diagnosed patients based on laboratory data, which we combine with information from their medical records. Patients who were still HCV‐positive when they left care are classified as eligible for retrieval if they are alive and currently residing in the Netherlands. They are subsequently invited by letter to an outpatient clinic of their choice after their current address is verified through municipality records or general practitioners. Data will be collected on patient and disease characteristics of patients who sign informed consent.

What we have learned since the start of CELINE in 2018 is that retrieval is feasible when conducted by a dedicated team. The project gives great insight into our care cascade and gives vital information for our hepatitis elimination plan. The nationwide approach ensures that retrieval is done to the same standards in each participating centre. Identification of LTFU patients and ensuring they adhere to their clinic appointments are the most time‐consuming. This is why we advise that a dedicated team, rather than individual clinicians, should execute these tasks.

## 13 INCLUDE RETRIEVAL IN STANDARD CARE

Ideally, micro‐elimination through retrieval should become standard of care. This concept is not (yet) mentioned in any guidelines or elimination plans, but deserves attention since it can contribute to HCV elimination. Retrieval could be done yearly, to reduce workload, and requires close collaboration between microbiologists/virologists, infectious disease specialists, hepatologists, hepatitis nurses and other parties such as addiction care medicine, prisons, public health institutes and/or general practitioners. Each centre could form a multidisciplinary team led by a dedicated retrieval coordinator, for example a hepatitis nurse. This coordinator could check the care cascade of all people who had a positive HCV test result in the previous year. The team could subsequently develop a multidisciplinary approach to retrieve LTFU patients. Patient‐centred care is key in retrieving LTFU patients.

## 14 ENSURING RETAINMENT IN CARE

Efforts should be made to retain LTFU and non‐LTFU patients in care. The cascade of care should be simplified as much as possible, as is stated in the call to action from the American Association for the Study of Liver Diseases (AASLD), the European Association for the Study of the Liver (EASL), the Asian Pacific Association for the Study of the Liver (APASL) and the Latin American Association for the Study of the Liver (ALEH), in partnership with the Clinton Health Access Initiative (CHAI).^28^ Pre‐treatment diagnostic assessment should be performed in one appointment. Treatment should be offered to all RNA‐positive patients. Patients should be treated using pangenotypic regimens, making genotyping beforehand obsolete. Monitoring during treatment should be kept to a minimum. Care should be decentralized and/or integrated within other disease programmes as much as possible. Task‐sharing between HCV specialists and other healthcare workers should be encouraged. Patients should be educated about the risk of re‐infection. Lastly, some patients should be retained in post‐SVR care, according to guidelines. This includes patients with a continuing risk of developing HCC, such as patients with advanced fibrosis (METAVIR score F3) or cirrhosis (F4) or patients with other risk factors such as excessive alcohol drinking, obesity and/or type 2 diabetes, but also patients with persisting abnormal liver tests that could indicate other causes of liver disease. These efforts can contribute to retainment in care and can therefore contribute to HCV elimination.

## 15 CONCLUSION

LTFU is a problem in each step in the HCV care cascade, even in the current era where highly effective treatments are available and where it has been possible to simplify the cascade of care. HCV can be micro‐eliminated in the LTFU population through retrieval. We present an example of nationwide retrieval in the Netherlands, which shows retrieval is feasible and can contribute to HCV elimination. We propose that micro‐elimination through retrieval becomes standard of care on the road to viral hepatitis elimination. Furthermore, efforts to retain patients in care should be implemented in daily clinical practice.

## Supporting information

Supplementary Material
