# Supplementary material for: Loss to follow‐up in the hepatitis C care cascade: A substantial problem but opportunity for micro‐elimination
Source: J Viral Hepat. 2020 Sep 22;27(12):1270–83. doi: 10.1111/jvh.13399 (PMC7693174; doi:10.1111/jvh.13399)
Supplement: Supplementary file 1 — Supplementary Material [file JVH-27-1270-s001.docx]

**Supplementary file 1**: Studies on the HCV cascade of care in the DAA era.

1. Zucker J, Aaron JG, Feller DJ, Slowikowski J, Evans H, Scherer ML, et al. Development and Validation of an Electronic Medical Record-Based Algorithm to Identify Patient Milestones in the Hepatitis C Virus Care Cascade. Open Forum Infect Dis. 2018;5(7):ofy153.

2. Assoumou SA, Wang J, Nolen S, Eftekhari Yazdi G, Mayer KH, Puro J, et al. HCV Testing and Treatment in a National Sample of US Federally Qualified Health Centers during the Opioid Epidemic. J Gen Intern Med. 2020.

3. Al-Khazraji A, Patel I, Saleh M, Ashraf A, Lieber J, Malik R. Identifying Barriers to the Treatment of Chronic Hepatitis C Infection. Dig Dis. 2020;38(1):46-52.

4. Moore MS, Bocour A, Laraque F, Winters A. A Surveillance-Based Hepatitis C Care Cascade, New York City, 2017. Public Health Rep. 2018;133(4):497-501.

5. Nguyen P, Vutien P, Hoang J, Trinh S, Le A, Yasukawa LA, et al. Barriers to care for chronic hepatitis C in the direct-acting antiviral era: a single-centre experience. BMJ Open Gastroenterol. 2017;4(1):e000181.

6. Marshall MC, Herrera JL. Lack of Patient Compliance in Real-World Practice Negatively Affects Sustained Viral Response Rates to Direct Acting Agent Therapy for Hepatitis C. Dig Dis Sci. 2018;63(12):3228-32.

7. Christensen S, Buggisch P, Mauss S, Böker KHW, Schott E, Klinker H, et al. Direct-acting antiviral treatment of chronic HCV-infected patients on opioid substitution therapy: Still a concern in clinical practice? Addiction. 2018;113(5):868-82.

8. Falade-Nwulia O, Sutcliffe CG, Mehta SH, Moon J, Chander G, Keruly J, et al. Hepatitis C Elimination in People With HIV Is Contingent on Closing Gaps in the HIV Continuum. Open Forum Infect Dis. 2019;6(10):ofz426.

9. Saris J, van den Berk G, Moha DA, van der Meer J, Brinkman K, van der Valk M. Successful implementation of hepatitis C virus treatment in two large HIV clinics in Amsterdam: hepatitis C virus treatment cascade of care. Aids. 2017;31(12):1779-80.

10. Kronfli N, Nitulescu R, Cox J, Moodie EE, Wong A, Cooper C, et al. Previous incarceration impacts access to hepatitis C virus (HCV) treatment among HIV-HCV co-infected patients in Canada. J Int AIDS Soc. 2018;21(11):e25197.

11. Adekunle RO, DeSilva K, Cartwright EJ. Hepatitis C Care Continuum in a Human Immunodeficiency Virus (HIV) Positive Cohort: Data From the HIV Atlanta Veterans Affairs Cohort Study. Open Forum Infect Dis. 2020;7(4):ofaa085.

12. Haridy J, Wigg A, Muller K, Ramachandran J, Tilley E, Waddell V, et al. Real-world outcomes of unrestricted direct-acting antiviral treatment for hepatitis C in Australia: The South Australian statewide experience. J Viral Hepat. 2018;25(11):1287-97.

13. Sølund C, Hallager S, Pedersen MS, Fahnøe U, Ernst A, Krarup HB, et al. Direct acting antiviral treatment of chronic hepatitis C in Denmark: factors associated with and barriers to treatment initiation. Scand J Gastroenterol. 2018;53(7):849-56.

14. Darvishian M, Wong S, Binka M, Yu A, Ramji A, Yoshida EM, et al. Loss to follow-up: A significant barrier in the treatment cascade with direct-acting therapies. J Viral Hepat. 2020;27(3):243-60.

15. Cachay ER, Mena A, Morano L, Benitez L, Maida I, Ballard C, et al. Predictors of Hepatitis C Treatment Failure After Using Direct-Acting Antivirals in People Living With Human Immunodeficiency Virus. Open Forum Infect Dis. 2019;6(3):ofz070.

16. Scaglione V, Mazzitelli M, Costa C, Pisani V, Greco G, Serapide F, et al. Virological and Clinical Outcome of DAA Containing Regimens in a Cohort of Patients in Calabria Region (Southern Italy). Medicina (Kaunas). 2020;56(3).

17. Boerekamps A, Newsum AM, Smit C, Arends JE, Richter C, Reiss P, et al. High Treatment Uptake in Human Immunodeficiency Virus/Hepatitis C Virus-Coinfected Patients After Unrestricted Access to Direct-Acting Antivirals in the Netherlands. Clin Infect Dis. 2018;66(9):1352-9.

18. Adamson PC, Miceli J, Shiferaw B, Villanueva MS, Canterino JE. A Colocalized Hepatitis C Virus Clinic in a Primary Care Practice Improves Linkage to Care in a High Prevalence Population. Am J Med. 2020.

19. Dever JB, Ducom JH, Ma A, Nguyen J, Liu L, Herrin A, et al. Engagement in Care of High-Risk Hepatitis C Patients with Interferon-Free Direct-Acting Antiviral Therapies. Dig Dis Sci. 2017;62(6):1472-9.

20. Trooskin SB, Poceta J, Towey CM, Yolken A, Rose JS, Luqman NL, et al. Results from a Geographically Focused, Community-Based HCV Screening, Linkage-to-Care and Patient Navigation Program. J Gen Intern Med. 2015;30(7):950-7.

21. Coyle C, Moorman AC, Bartholomew T, Klein G, Kwakwa H, Mehta SH, et al. The Hepatitis C Virus Care Continuum: Linkage to Hepatitis C Virus Care and Treatment Among Patients at an Urban Health Network, Philadelphia, PA. Hepatology. 2019;70(2):476-86.

22. Bajis S, Grebely J, Cooper L, Smith J, Owen G, Chudleigh A, et al. Hepatitis C virus testing, liver disease assessment and direct-acting antiviral treatment uptake and outcomes in a service for people who are homeless in Sydney, Australia: The LiveRLife homelessness study. J Viral Hepat. 2019;26(8):969-79.

23. Waked I, Esmat G, Elsharkawy A, El-Serafy M, Abdel-Razek W, Ghalab R, et al. Screening and Treatment Program to Eliminate Hepatitis C in Egypt. New England Journal of Medicine. 2020;382(12):1166-74.

24. Khalid GG, Kyaw KWY, Bousquet C, Auat R, Donchuk D, Trickey A, et al. From risk to care: the hepatitis C screening and diagnostic cascade in a primary health care clinic in Karachi, Pakistan-a cohort study. Int Health. 2020;12(1):19-27.

25. Hsieh YH, Signer D, Patel AV, Viertel V, Saheed M, Irvin R, et al. Advanced liver fibrosis and care continuum in emergency department patients with chronic hepatitis C. Am J Emerg Med. 2019;37(2):286-90.

26. Zuckerman A, Douglas A, Nwosu S, Choi L, Chastain C. Increasing success and evolving barriers in the hepatitis C cascade of care during the direct acting antiviral era. PLoS One. 2018;13(6):e0199174.

27. Evans H, Balasegaram S, Douthwaite S, Hunter L, Kulasegaram R, Wong T, et al. An innovative approach to increase viral hepatitis diagnoses and linkage to care using opt-out testing and an integrated care pathway in a London Emergency Department. PLoS One. 2018;13(7):e0198520.

28. Benitez TM, Fernando SM, Amini C, Saab S. Geographically Focused Collocated Hepatitis C Screening and Treatment in Los Angeles's Skid Row. Dig Dis Sci. 2020.

29. Falade-Nwulia O, Ward KM, McCormick S, Mehta SH, Pitts SR, Katz S, et al. Network-based recruitment of people who inject drugs for hepatitis C testing and linkage to care. J Viral Hepat. 2020.

30. Bajis S, Grebely J, Hajarizadeh B, Applegate T, Marshall AD, Ellen Harrod M, et al. Hepatitis C virus testing, liver disease assessment and treatment uptake among people who inject drugs pre- and post-universal access to direct-acting antiviral treatment in Australia: The LiveRLife study. J Viral Hepat. 2020;27(3):281-93.

31. Alimohammadi A, Holeksa J, Parsons R, Yung R, Amiri N, Truong D, et al. Diagnosis and treatment of hepatitis C virus infection: a tool for engagement with people who inject drugs in Vancouver's Downtown Eastside. Can Liv J. 2018;1(2):14-33.

32. Harrison GI, Murray K, Gore R, Lee P, Sreedharan A, Richardson P, et al. The Hepatitis C Awareness Through to Treatment (HepCATT) study: improving the cascade of care for hepatitis C virus-infected people who inject drugs in England. Addiction. 2019;114(6):1113-22.

33. Capileno YA, Van den Bergh R, Donchunk D, Hinderaker SG, Hamid S, Auat R, et al. Management of chronic Hepatitis C at a primary health clinic in the high-burden context of Karachi, Pakistan. PLoS One. 2017;12(4):e0175562.

34. Cooper CL, Hatashita H, Corsi DJ, Parmar P, Corrin R, Garber G. Direct-Acting Antiviral Therapy Outcomes in Canadian Chronic Hepatitis C Telemedicine Patients. Ann Hepatol. 2017;16(6):874-80.

35. Shiha G, Metwally AM, Soliman R, Elbasiony M, Mikhail NNH, Easterbrook P. An educate, test, and treat programme towards elimination of hepatitis C infection in Egypt: a community-based demonstration project. Lancet Gastroenterol Hepatol. 2018;3(11):778-89.

36. Ford MM, Johnson N, Desai P, Rude E, Laraque F. From Care to Cure: Demonstrating a Model of Clinical Patient Navigation for Hepatitis C Care and Treatment in High-Need Patients. Clin Infect Dis. 2017;64(5):685-91.

37. Bartholomew TS, Grosgebauer K, Huynh K, Cos T. Integration of Hepatitis C Treatment in a Primary care Federally Qualified Health Center; Philadelphia, Pennsylvania, 2015-2017. Infect Dis (Auckl). 2019;12:1178633719841381.

38. Wade AJ, McCormack A, Roder C, McDonald K, Davies M, Scott N, et al. Aiming for elimination: Outcomes of a consultation pathway supporting regional general practitioners to prescribe direct-acting antiviral therapy for hepatitis C. J Viral Hepat. 2018;25(9):1089-98.

39. Mendizabal M, Ridruejo E, Ceballos S, Sixto M, Billordo A, Gadea C, et al. The ECHO model proved to be a useful tool to increase clinicians' self-effectiveness for care of patients with Hepatitis C in Argentina. J Viral Hepat. 2019;26(11):1284-92.

40. Wade AJ, Doyle JS, Gane E, Stedman C, Draper B, Iser D, et al. Outcomes of Treatment for Hepatitis C in Primary Care, Compared to Hospital-based Care: A Randomized, Controlled Trial in People Who Inject Drugs. Clin Infect Dis. 2020;70(9):1900-6.

41. O'Sullivan M, Jones A-M, Gage H, Jordan J, MacPepple E, Williams H, et al. ITTREAT (Integrated Community Test - Stage - TREAT) Hepatitis C service for people who use drugs: Real-world outcomes. Liver International. 2020;n/a(n/a).

42. Norton BL, Fleming J, Bachhuber MA, Steinman M, DeLuca J, Cunningham CO, et al. High HCV cure rates for people who use drugs treated with direct acting antiviral therapy at an urban primary care clinic. Int J Drug Policy. 2017;47:196-201.

43. Kattakuzhy S, Gross C, Emmanuel B, Teferi G, Jenkins V, Silk R, et al. Expansion of Treatment for Hepatitis C Virus Infection by Task Shifting to Community-Based Nonspecialist Providers: A Nonrandomized Clinical Trial. Ann Intern Med. 2017;167(5):311-8.

44. Morris L, Smirnov A, Kvassay A, Leslie E, Kavanagh R, Alexander N, et al. Initial outcomes of integrated community-based hepatitis C treatment for people who inject drugs: Findings from the Queensland Injectors' Health Network. Int J Drug Policy. 2017;47:216-20.

45. Cachay ER, Hill L, Torriani F, Ballard C, Grelotti D, Aquino A, et al. Predictors of Missed Hepatitis C Intake Appointments and Failure to Establish Hepatitis C Care Among Patients Living With HIV. Open Forum Infect Dis. 2018;5(7):ofy173.

46. Kim NJ, Locke CJ, Park H, Magee C, Bacchetti P, Khalili M. Race and Hepatitis C Care Continuum in an Underserved Birth Cohort. J Gen Intern Med. 2019;34(10):2005-13.

47. Starbird LE, Budhathoki C, Han HR, Sulkowski MS, Reynolds NR, Farley JE. Nurse case management to improve the hepatitis C care continuum in HIV co-infection: Results of a randomized controlled trial. J Viral Hepat. 2020;27(4):376-86.

48. Ward KM, Falade-Nwulia O, Moon J, Sutcliffe CG, Brinkley S, Haselhuhn T, et al. A Randomized Controlled Trial of Cash Incentives or Peer Support to Increase HCV Treatment for Persons With HIV Who Use Drugs: The CHAMPS Study. Open Forum Infect Dis. 2019;6(4):ofz166.

49. Rizk C, Miceli J, Shiferaw B, Malinis M, Barakat L, Ogbuagu O, et al. Implementing a Comprehensive HCV Clinic within an HIV Clinic: A Model of Care for HCV Micro-elimination. Open Forum Infect Dis. 2019;6(10).

50. Carvalho-Louro DM, Soares EB, Trevizoli JE, Marra TMG, da Cunha ALR, Rodrigues MP, et al. Hepatitis C screening, diagnosis, and cascade of care among people aged > 40 years in Brasilia, Brazil. BMC Infect Dis. 2020;20(1):114.

51. Averhoff F, Shadaker S, Gamkrelidze A, Kuchuloria T, Gvinjilia L, Getia V, et al. Progress and challenges of a pioneering hepatitis C elimination program in the country of Georgia. J Hepatol. 2020;72(4):680-7.

52. Hutton J, Doyle J, Zordan R, Weiland T, Cocco A, Howell J, et al. Point-of-care Hepatitis C virus testing and linkage to treatment in an Australian inner-city emergency department. Int J Drug Policy. 2019;72:84-90.

53. Chiong F, Post J. Opportunistic assessment and treatment of people with hepatitis C virus infection admitted to hospital for other reasons: A prospective cohort study. Int J Drug Policy. 2019;65:50-5.

54. Read P, Lothian R, Chronister K, Gilliver R, Kearley J, Dore GJ, et al. Delivering direct acting antiviral therapy for hepatitis C to highly marginalised and current drug injecting populations in a targeted primary health care setting. Int J Drug Policy. 2017;47:209-15.

55. Koren DE, Zuckerman A, Teply R, Nabulsi NA, Lee TA, Martin MT. Expanding Hepatitis C Virus Care and Cure: National Experience Using a Clinical Pharmacist-Driven Model. Open Forum Infect Dis. 2019;6(7).

56. Nouch S, Gallagher L, Erickson M, Elbaharia R, Zhang W, Wang L, et al. Factors associated with lost to follow-up after hepatitis C treatment delivered by primary care teams in an inner-city multi-site program, Vancouver, Canada. Int J Drug Policy. 2018;59:76-84.

57. White L, Azzam A, Burrage L, Orme C, Kay B, Higgins S, et al. Facilitating treatment of HCV in primary care in regional Australia: closing the access gap. Frontline Gastroenterol. 2019;10(3):210-6.

58. Koustenis KR, Anagnostou O, Kranidioti H, Vasileiadi S, Antonakaki P, Koutli E, et al. Direct-acting antiviral treatment for chronic hepatitis C in people who use drugs in a real-world setting. Ann Gastroenterol. 2020;33(2):195-201.

59. McMahon BJ, Townshend-Bulson L, Homan C, Gounder P, Barbour Y, Hewitt A, et al. Cascade of Care for Alaska Native People with Chronic Hepatitis C Virus Infection: Statewide Program with High Linkage to Care. Clin Infect Dis. 2019.

60. Sherbuk JE, McManus KA, Kemp Knick T, Canan CE, Flickinger T, Dillingham R. Disparities in Hepatitis C Linkage to Care in the Direct Acting Antiviral Era: Findings From a Referral Clinic With an Embedded Nurse Navigator Model. Front Public Health. 2019;7:362.

61. Francheville JW, Rankin R, Beck J, Hoare C, Materniak S, German G, et al. Early Successes in an Open Access, Provincially Funded Hepatitis C Treatment Program in Prince Edward Island. Ann Hepatol. 2018;17(2):223-31.

62. Mohsen W, Chan P, Whelan M, Glass A, Mouton M, Young E, et al. Hepatitis C treatment for difficult to access populations: can telementoring (as distinct from telemedicine) help? Intern Med J. 2019;49(3):351-7.
